# Reporting of dog-assisted intervention trials: extension of the SPIRIT 2025 and CONSORT 2025 statement

**DOI:** 10.1186/s12874-026-02848-7

**Published:** 2026-04-20

**Authors:** Sophie S. Hall, Emily Shoesmith, Evgenia Riga, Daniel S. Mills, Selina Gibsone, Dean McMillan, Qi Wu, Chris Clarke, Elena Ratschen

**Affiliations:** 1 Nottingham Clinical Trials Unit, School of Medicine, University of Nottingham, Nottingham, UK; 2 Department of Health Sciences, University of York, York, UK; 3 Department of Life Sciences, University of Lincoln, Lincoln, UK; 4 Dogs for Good, Banbury, UK; 5 Tees, Esk and Wear Valleys, Foss Park Hospital, NHS Foundation Trust, York, UK

**Keywords:** SPIRIT, CONSORT, Dog-Assisted Interventions, Reporting guidelines

## Abstract

**Background:**

Transparent reporting of dog-assisted intervention (DAI) research is important to support critical appraisal and interpretation of study designs and results. DAIs, in which a dog plays a role in the delivery of therapeutic intervention to enhance the wellbeing and support of individuals, have specific methodological considerations. However, reporting of DAI trial designs and results are suboptimal, preventing the development of a robust evidence base to guide research and practice in this area.

**Aim:**

To develop consensus-based extensions to the Standard Protocol Items: Recommendations for Interventional Trials (SPIRIT) 2025 and Consolidated Standards of Reporting Trials (CONSORT) 2025 Statement for DAI research.

**Methods:**

Using the Enhancing the Quality and Transparency of Health Research (EQUATOR) methodological framework a four-phase, consensus-based approach was used, including: 1) a systematic review of DAI Randomised Controlled Trials (RCTs); 2) a three-round Delphi survey (*n* = 83; *n* = 74; *n* = 71 respectively); 3) consensus workshops with experts in DAI research and/or clinical trial design and reporting (*n* = 7), and 4) checklist piloting and finalisation.

**Results:**

The systematic review (phase 1) identified 85 shortcomings in reporting DAI (44 applied to SPIRIT and 41 to CONSORT) to include in the extension development. These shortcomings formed the basis of the items on the three-round DELPHI survey that followed. In the end, 96 extension items reached consensus for consideration (phase 2). The concepts were further refined and translated into specific extension item wording through a comprehensive review process led by the core team in collaboration with leading DAI experts (phase 3). This resulted in 71 extensions for DAI research reporting (36 applied to SPIRIT and 35 applied to CONSORT). The items were piloted (phase 4), and appropriate examples sought for a future explanation and elaboration document (not reported here).

**Conclusion:**

The new SPIRIT and CONSORT extensions are hoped to enhance the reporting, as well as conduct, understanding and interpretation of DAI trials.

**Supplementary Information:**

The online version contains supplementary material available at 10.1186/s12874-026-02848-7.

## Background

Animal-Assisted Interventions (AAIs) are increasingly implemented in healthcare settings as a form of complementary or adjunctive therapy to improve mental health outcomes in the UK National Health Service (NHS) settings [1, 2]. Dogs are the most common animal species involved in AAIs, perhaps due to humans’ desire to form emotional connections with them, and their receptiveness to human cues and behavioural training [3, 4]. Dog-Assisted Interventions (DAIs) involve dogs in health, education and social services contexts for therapeutic or otherwise ameliorative purposes. DAIs include dog-assisted therapy, which is goal-orientated, structured, documented, and delivered by trained professionals; and dog-assisted activities, which are also goal-orientated, but typically based on spontaneous interaction, delivered usually by volunteers and non-specialist trained dogs.

The presence of a dog may enhance standard therapy processes by accelerating rapport building between patient and therapist [5, 6] and improving therapeutic alliance [7, 8]. Moreover, a patient may be more motivated to engage in therapy in the presence of a dog [9], which can increase treatment satisfaction and ultimately adherence [10–12]. These benefits have the potential to improve healthcare efficiency, speeding up time to a successful outcome and reducing missed appointments, but a stronger evidence base is required to enable realisation of the true benefits [2].

Randomised Controlled Trials (RCTs) are considered the gold standard way to evaluate healthcare innovations. However, due the complex nature of DAIs, whereby an animal and third parties (e.g. dog handlers) are added into the intervention, DAIs require unique considerations in the design, conduct and reporting of RCTs in this area. Despite the growth of RCTs in the area [13–16], evidence syntheses [17–22] have unanimously pointed out common methodological flaws and lack of rigour in study design. DAI are increasingly used within health and social care settings, particularly in high-middle income countries (e.g. UK, USA, Australia), where structured therapy-dog programmes operate in hospitals, rehabilitation centres, and care homes. Organisations such as Pets As Therapy, Dogs for Good (UK), Pet Partners (USA) and Delta Therapy Dogs (Australia) illustrate the growing institutional integration of these programmes. As DAIs are increasingly adopted in practice, this poor-quality evidence base raises real risks for both humans and animals involved. Common issues include lack of statistical power; large heterogeneity of outcome measures; an absence of manualised intervention protocols (including details relating to the animal partner, including species, procedures to choose/train/risk assess) and well-designed control conditions; as well as the use of ambiguous terminologies [21–24]. In addition, the design of a DAI RCT must consider the welfare and safety for the patient, dog, and therapist/handler. Design issues are further compounded by poor reporting, limiting reproducibility, and comparability [22, 24, 25]. The complex nature of DAIs requires consideration of that exceeds current design and reporting practice in the field [26], and there are calls to improve this [2].

In response to inadequate reporting of RCTs in general, the Consolidated Standard of Reporting Trials (CONSORT) was published and subsequently updated [27–29], with a recent revision published in 2025 [30]. The guidance includes a checklist of minimum items that should be included when reporting the results of an RCT. Similarly, Standard Protocol Items: Recommendations for Interventional Trials (SPIRIT) guidelines were first published in 2013 to improve the completeness and transparency of protocols for clinical trials [31], with a recent revision published in 2025 [32]. Extensions guidelines have been crafted to expand the original SPIRIT and CONSORT statements. These extensions offer further instructions for reporting across specific trial designs, data types, and interventions [33–52] which are not covered in the original SPIRIT and CONSORT statements.

Here, an extension of the CONSORT 2025 and SPIRIT 2025 checklists for the reporting of DAIs is presented with the aim of improving reporting standards in this field. Definitions in human-animal interaction research are used inter-changeably and there is no internationally accepted terminology of classifications. As such, a glossary of key terms commonly used by authors in the field are provided in Table 1.

**Table 1:** Glossary of key terms specific to DAIs

Term	Acronym	Description
Animal-Assisted Intervention	AAI	An umbrella term for interventions in health, social care, education and other contexts where animals are involved for therapeutic or other wellbeing-related purposes
Dog-Assisted Intervention	DAI	An umbrella term for interventions in health, social care, education and other contexts where dogs are involved for therapeutic or other wellbeing-related purposes
Dog-Assisted Therapy	DAT	A sub-category of DAIs, this refers to a goal-orientated therapy that is structured and delivered by trained and certified dog handler teams
Dog-Assisted Activities	DAA	A sub-category of DAIs, this refers to activities that are less tailored to a specific goal but typically aim to improve overall wellbeing. Interactions are usually spontaneous, and the activities are often delivered by volunteers
Dog-Assisted Education	DAE	A sub-category of DAIs, this refers to the involvement of qualified educational professionals and trained/certified dog handler teams in educational settings to support the learning environment
Dog-Assisted Counselling	DAC	A sub-category of DAIs, this refers to a therapeutic approach that incorporates the presence of trained dog handler teams in to counselling sessions
Dog-Assisted Services	DAS	New terminology used to replace ‘DAI’ as the umbrella term for the full spectrum of practices in this area. Binder et al. [53] define DAS as ‘mediated, guided or facilitator-led practices, programs and human services that incorporate specially qualified animals into therapeutic, educational, supportive and/or ameliorative processes aimed at enhancing the well-being of humans while ensuring the welfare of the animals involved in these practices.’*
Dog-Assisted Treatment	DATx	A sub-category of the new ‘DAS’ terminology, replacing Dog-Assisted Therapy. Binder et al. [53] define DATx ‘a class of mental or physical health professional treatment modalities for which the integration of animals, directly or indirectly, is a critical component of the treatment approach of the professional.’ Binder et al. state that a competent and ethical DATx professional should be trained and supervised first in their licensed/credentialed profession (e.g., counselling, occupational therapy) and then in the specialised area of treatment being provided (e.g., animal-assisted psychotherapy, animal-assisted occupational therapy, animal-assisted speech therapy)
Dog-Assisted Support Programs	DASP	A sub-category of the new ‘DAS’ terminology, replacing Dog-Assisted Activities. Binder et al. [53] refer to DASP as ‘programs in which animals are engaged, directly or indirectly, in activities aimed at supporting and enhancing the well-being of humans. These programs may have aims that include increased motivation, prevention of loneliness and isolation, reduction of tension and anxiety, distraction from difficult situations, or emotional comfort. In the case of animal visitation services, the human service providers and animal specialists may be volunteer handlers with related knowledge in animal behaviour and training at the species, breed, and individual levels. The activities may or may not be documented, depending on the goals and demands of the specific program being carried out.’

^*^We discussed using the term DAS throughout the development of these extensions. However, our expert group believed that whilst readers should be aware of the potential change in terminology, this has yet to be widely accepted by practitioners. In addition, feedback from stakeholders suggests potential problems with adopting the term DAS internationally (as it translates poorly into some languages)

## Methods

The development of the SPIRIT and CONSORT extension occurred in parallel, based on SPIRIT 2013 and CONSORT 2010 guidelines, which were the most recent versions at the time of the Delphi process. Following the subsequent publication of SPIRIT 2025 and CONSORT 2025, all developed extensions were mapped against the updated guidelines to ensure continued relevance and alignment. Although the 2025 versions introduced refinements and greater consistency between SPIRIT and CONSORT, the core underlying concepts remained largely unchanged (e.g., ‘Identification as randomised trial in the title’ became ‘identification as a randomised trial’. The addition and deletion of new items did not affect our extensions. Consequently, no modifications to the content or wording of the developed extensions were required, and the overall findings and recommendations of this work were not affected. Patient and Public Involvement (PPI) members contributed through regular meetings and consultation sessions to ensure the guidance reflected priorities in DAI research and also provided independent verification of themapping process.

This study was registered with the Enhancing the QUAlity and Transparency Of health Research (EQUATOR) network [54] and followed their methods for developing healthcare reporting guidelines (20). The study protocol was published prior to commencement of the research activities on Open Science Framework (388928). Ethical approval was sought from the University of York’s Health Sciences Research Governance Committee (HSRGC/2023/586/E). Participants gave informed consent. Development of the extensions followed a four-phase approach:

The first phase involved a systematic review of the literature to identify DAI studies on mental health and neurodevelopmental conditions (across the life span), using a RCT design, and extract key reporting considerations from the literature to develop a ‘long-list’ of SPIRIT and CONSORT extension items [22]. Reporting considerations were extracted separately from the analysis reported in the literature review and are highly relevant for the reporting of the comparatively fewer DAI trials for other health conditions.

In the second phase, a three-round Delphi survey was conducted to refine the long-list of items by removing those deemed less important and considering the addition of new items. The survey was hosted by Welphi; a platform commonly used to conduct Delphi surveys online. Participants were recruited for the Delphi process by identifying international experts in the research and delivery of DAIs and the development of reporting guidelines, drawing on the study team's contacts and the authors of relevant published work. The three rounds of the Delphi survey enabled participants to propose new items in the first-round while ensuring that all items were rated at least twice. Explanatory text was added to the items where applicable after feedback from piloting. In each round, participants were asked to rate the importance of including each concept in the final set of guidelines for both the SPIRIT and CONSORT extensions. There was an option for free text feedback on the items at the end of the survey. Items were presented and rated separately for SPIRIT and CONSORT extensions, even if the item used the same wording. Rating used a 9-point Likert scale: 1–3 = not important; 4–6 = important but not critical; 7–9 = critical. An "unable to rate" option was also provided, along with space for free text to explain ratings. After each survey round, the ratings for each item were tabulated and categorised as follows:

- Consensus in: ≥ 70% of participants scored 7–9, and < 15% scored 1–3.
- Consensus out: ≥ 70% of participants scored 1–3, and < 15% scored 7–9.
- No consensus: Any other outcome.

Quantitative data (percentages) were calculated using Excel formulas, and qualitative data (free-text comments) were reviewed and contextualised; both by EvR and reviewed by SSH and ES. Summary tables of the quantitative and qualitative data were produced and shared with the research team at the end of each round. Quantitative data were used to determine consensus on each item and qualitative data were informed the development of the accompanying explanation and elaboration document (published separately to this checklist paper).

The third phase of development consisted of two half-day online consensus workshops. Individuals identified by the study team as having expertise in DAI research and a track record of high-quality reporting were invited to participate in reviewing the items not at consensus, finalising the wording of those items at consensus to include in the extensions, and suggesting appropriate text and references for the Explanation and Elaboration (E&E) document. Three members of the core study team (SSH, ES, ER) attended each workshop. The two items which did not reach consensus in the Delphi survey were discussed by the workshop members at both workshops. Anonymised live voting was used to achieve consensus online, with a unanimous decision to keep both items in following minor rewording. The wording for each item at consensus was reviewed and refined as appropriate (see Supplementary Material 3). Minor agreed changes to item wording and other key decisions were recorded in a document shared live with attendees. ES and SSH updated the extension items as per the recorded actions. Following the workshops, attendees shared extracts and references for the E&E document with the study team. All contributions were made on a live document shared with all attendees and the study team.

Finally, the fourth ‘checklist finalisation’ phase involved iterative revisions and piloting of the extension items and drafting the supporting E&E statements between the study team. Members of the workshop were consulted when decisions could not be reached within the study team. After the release of the updated SPIRIT 2025 and CONSORT 2025, all extensions were mapped against, reviewed and agreed by our wider study advisory group (including clinicians, academics, and animal behaviourists).

## Results

### Phase one: systematic review

The systematic review identified 33 RCTs evaluating DAIs [22]. The mean proportion of adherence to the CONSORT statement was 48.6%. Data extracted from these articles produced 85 reporting recommendations (44 applicable to SPIRIT and 41 applicable to CONSORT). The review included published results of RCTs only, protocol papers were not included. However, similar to existing efforts in this area [55, 56], we identified reporting recommendations in the manuscripts which were applicable to both SPIRIT and CONSORT statements. Because protocols were not part of the systematic review, we conducted an additional targeted search of clinical trial databases (ClinicalTrials.gov and international Standard Registered Clinical/Social Study Number: ISRTCN), to identify relevant published protocols in the area (*n* = 8). These protocols were used to inform the development of candidate SPIRIT extension items, but they were not included in the systematic review analyses.

### Phase two: Delphi survey

The 85 reporting recommendations formed the items in the Delphi survey. The Delphi survey ran from April 2024 to September 2024, with 83, 74 and 71 participants completing rounds one, two and three respectively. Based on our final sample (*n* = 71, 86% completion rate from round one), the majority of participants’ main experience in AAI was as a researcher/academic (*n* = 50, 70%), followed by therapist/counsellor/clinician (*n* = 13, 18%). On average, participants had 11.15 years of experience in the field of AAIs, publishing 7.31 papers on average in this field (see Supplementary Material 1 for full demographic information).

After round one, from the 85 items which were presented for rating across the proposed SPIRIT and CONSORT extensions, 64/85 items (75%) met the criteria for ‘consensus in’ (SPIRIT: 34/44 (77%) CONSORT: 30/41 (90%)), 0/85 items (0%) met the criteria for ‘consensus out’ and 21/85 items (25%) did not reach consensus (SPIRIT: 10/21; CONSORT: 11/21). 13 new items were suggested by participants. After review by the study team, six items were added to round two, 16 items had further explanatory information added, and 18 items were edited to improve comprehension (see Supplementary Material 2).

After round two, from the 91 items which were presented for rating across the proposed SPIRIT and CONSORT extensions, 80/91 items (88%) met the criteria for ‘consensus in’ (SPIRIT: 45/48; CONSORT: 35/43), 0/91 items (0%) met the criteria for ‘consensus out’ and 11/91 items (12%) did not reach consensus (SPIRIT: 3/11; CONSORT: 8/11). The new items included in this round were presented in round three regardless of whether they had reached consensus in this round.

After round three, from the 16 items which were presented for rating across the proposed SPIRIT and CONSORT extensions, 14/16 items (88%) met the criteria for ‘consensus in’ (SPIRIT: 6/7; CONSORT: 8/9), 0/16 items (0%) met the criteria for ‘consensus out’ and 2/16 items (13%) did not reach consensus (SPIRIT: 1/2; CONSORT: 1/2).

### Phase three: consensus workshops

The consensus workshops were attended by a total of seven (male = 2; USA = 2, Europe = 2; UK = 3) experts (4 in workshop one, 3 in workshops two) with international recognition in the field of DAI research (*n* = 6) and/or clinical trial design and reporting (*n* = 1).

### Phase four: Checklist finalisation 

The checklists, as agreed upon in the consensus workshops, were iteratively reviewed by the core study team and elaboration text drafted. Examples of good reporting were sourced by the study team and members of the consensus workshop (these will be reported in a separate paper). During the process of mapping the drafted DAI extensions to the updated SPIRIT and CONSORT 2025 checklists, an item which was previously only presented in the SPIRIT DAI checklist was subsequently added to the CONSORT DAI checklist as well. This was due to the addition of CONSORT 12b (If applicable, eligibility criteria for sites and for individuals delivering the interventions (e.g., surgeons, physiotherapists) [30].

The finalised checklist and E&E document were pilot tested by four authors, three of whom were experienced in DAI research (SH, ES, ER), and one who had no prior experience (EvR). Each considered a different piece of published DAI research and used the checklists to outline key points in the study protocol (where available) and main results publication manuscript. This enabled the authors to reflect on the clarity of the item wording.

### Checklist items

As a result of the four-phase approach detailed above, a total of 71 DAI extension items were created, 36 applying to SPIRIT and 35 applying to CONSORT. All items were designed to extend as opposed to replace the original SPIRT 2025 (Table 2) or CONSORT 2025 item (see Table 3). Unless otherwise stated, the items presented below are intended for use as extensions to both SPIRIT and CONSORT items.

**Table 2:** Checklist for reporting of DAIs: extension of the SPIRIT 2025 statement

Section/topic	No	SPIRIT 2025 checklist item	Extension for DAI RCTs
*Administrative information*			
Title and structured summary	1a	Title stating the trial design, population, and interventions, with identification as a protocol	The species of animal involved (i.e., dog)
			Type of DAI (e.g., therapy, activity, education)
	1b	Structured summary of trial design and methods, including items from the World Health Organization Trial Registration Data Set	N/A
Protocol version	2	Version date and identifier	N/A
Roles and responsibilities	3a	Names, affiliations, and roles of protocol contributors	N/A
	3b	Name and contact information for the trial sponsor	N/A
	3c	Role of trial sponsor and funders in design, conduct, analysis, and reporting of trial; including any authority over these activities	N/A
	3d	Composition, roles, and responsibilities of the coordinating site, steering committee, endpoint adjudication committee, data management team, and other individuals or groups overseeing the trial, if applicable	Roles and responsibilities of individual(s) responsible for dog welfare
			State which DAI guidelines/code of practice are used if appropriate
			Name, accreditation status, non-/for-profit status of any animal-assisted intervention organisations involved in intervention (where applicable)
*Open Science*			
Trial registration	4	Name of trial registry, identifying number (with URL), and date of registration. If not yet registered, name of intended registry	N/A
Protocol and statistical analysis plan	5	Where the trial protocol and statistical analysis plan can be accessed	N/A
Data sharing	6	Where and how the individual de-identified participant data (including data dictionary), statistical code, and any other materials will be accessible	N/A
Funding and conflicts of interest	7a	Sources of funding and other support (for example, supply of drugs)	N/A
	7b	Financial and other conflicts of interest for principal investigators and steering committee member	N/A
Dissemination policy	8	Plans to communicate trial results to participants, healthcare professionals, the public, and other relevant groups (for example, reporting in trial registry, plain language summary, publication)	N/A
*Introduction*			
Background and rationale	9a	Scientific background and rationale, including summary of relevant studies (published and unpublished) examining benefits and harms for each intervention	Scientific background and rationale for including a DAI
			Description of proposed mechanism(s), model(s) or theories describing the potential impact of the DAI, if applicable
	9b	Explanation for choice of comparator	N/A
Objectives	10	Specific objectives related to benefits and harms	Specific objectives or hypotheses related to the DAI
*Methods: Patient and public involvement, trial design*			
Patient and public involvement	11	Details of, or plans for, patient or public involvement in the design, conduct, and reporting of the trial	N/A
Trial design	12	Description of trial design including type of trial (for example, parallel group, crossover), allocation ratio, and framework (for example, superiority, equivalence, non-inferiority, exploratory)	N/A
*Methods: Participants, interventions and outcomes*			
Trial setting	13	Settings (for example, community, hospital) and locations (for example, countries, sites) where the trial will be conducted	Description of how considerations related to dog welfare are incorporated in the study context and settings
			Description of the characteristics of study settings and the DAI that may affect dogs (e.g., room size, noises, smells, lighting)
			Description of how the suitability of the dog for the DAI in the given study context and environment is ensured
Eligibility criteria	14a	Eligibility criteria for participants	Eligibility criteria relevant to engaging/interacting with dog, if any (e.g., dog phobia, allergies, history of dog abuse)
	14b	If applicable, eligibility criteria for sites and for individuals who will deliver the interventions (for example, surgeons, physiotherapists)	Description of any criteria for sites to ensure dog welfare requirements can be met
			Selection criteria for the dog(s) involved in the intervention and justification for these
			Description of relevant training and education undertaken by all parties that is pertinent to their role in the intervention (e.g., participant vs. dog handler). This may include training on dog welfare, safety and intervention-specific content
Intervention and comparator	15a	Intervention and comparator with sufficient details to allow replication including how, when, and by whom they will be administered. If relevant, where additional materials describing the intervention and comparator (for example, intervention manual) can be accessed	Description of DAI goal and content
			Description of the proposed duration and frequency of DAI including, where possible, justification
			Description of tasks and roles of each individual in the DAI team (i.e., dog, dog handler, other trained professional) including details of participant-dog interactions
			Where possible, consideration of suitability/tailoring of DAI under EDI aspects (e.g., severity of illness)
			Rationale for and description of the comparator group
	15b	Criteria for discontinuing or modifying allocated intervention/comparator for a trial participant (for example, drug dose change in response to harms, participant request, or improving/worsening disease)	Criteria and/or processes for discontinuing or modifying the intervention based on dog and/or handler responses
	15c	Strategies to improve adherence to intervention/comparator protocols, if applicable, and any procedures for monitoring adherence (for example, drug tablet return, sessions attended)	N/A
	15d	Concomitant care that is permitted or prohibited during the trial	Description of whether and how potential concomitant human–dog interactions (e.g., with pet dog) are controlled for
Outcomes	16	Primary and secondary outcomes, including the specific measurement variable (for example, systolic blood pressure), analysis metric (for example, change from baseline, final value, time to event), method of aggregation (for example, median, proportion), and time point for each outcome	Outcome measures relevant to the proposed pathway of action of the DAI
			Where applicable, justify the use of unblinded outcome measures in relation to the DAI
Harms	17	How harms are defined and assessed (for example, systematically, non-systematically)	Observations/measures relevant to the impact of the intervention on the dog(s)
Participant timeline	18	Time schedule of enrolment, interventions (including any run-ins and washouts), assessments, and visits for participants. A schematic diagram is highly recommended	Time schedule of any required participant-dog training/familiarity sessions prior to commencement of the intervention
Sample size	19	How sample size was determined, including all assumptions supporting the sample size calculation	Description of capacity of dog-handler teams required to safely and effectively deliver the intervention
Recruitment	20	Strategies for achieving adequate participant enrolment to reach target sample size	N/A
*Methods: Assignment of interventions*			
Sequence generation	21a	Who will generate the random allocation sequence, and the method used	If possible, describe the process of matching dog-handler teams (dog & handler) to participants, including the individual/team responsible for this process, where relevant
	21b	Type of randomization (simple or restricted) and details of any factors for stratification. To reduce predictability of a random sequence, other details of any planned restriction (for example, blocking) should be provided in a separate document that is unavailable to those who enrol participants or assign interventions	Description of whether concomitant interactions with dogs (e.g., interactions with pet/other dogs) is included as a stratification variable
Allocation concealment mechanism	22	Mechanism used to implement the random allocation sequence (for example, central computer/telephone; sequentially numbered, opaque, sealed containers), describing any steps to conceal the sequence until interventions are assigned	N/A
Implementation	23	Whether the personnel who will enrol and those who will assign participants to the interventions will have access to the random allocation sequence	N/A
Blinding	24a	Who will be blinded after assignment to interventions (for example, participants, care providers, outcome assessors, data analysts)	Description and justification of whether trained professional(s) and dog handler(s) are blinded to the outcomes and how
	24b	If blinded, how blinding will be achieved and description of the similarity of interventions	N/A
	24c	If blinded, circumstances under which unblinding is permissible, and procedure for revealing a participant’s allocated intervention during the trial	N/A
*Methods: Data collection, management, and analysis*			
Data collection methods	25a	Plans for assessment and collection of trial data, including any related processes to promote data quality (for example, duplicate measurements, training of assessors) and a description of trial instruments (for example, questionnaires, laboratory tests) along with their reliability and validity, if known. Reference to where data collection forms can be accessed, if not in the protocol	N/A
	25b	Plans to promote participant retention and complete follow-up, including list of any outcome data to be collected for participants who discontinue or deviate from intervention protocols	N/A
Data management	26	Plans for data entry, coding, security, and storage, including any related processes to promote data quality (for example, double data entry; range checks for data values). Reference to where details of data management procedures can be accessed, if not in the protocol	N/A
Statistical methods	27a	Statistical methods used to compare groups for primary and secondary outcomes, including harms	N/A
	27b	Definition of who will be included in each analysis (for example, all randomized participants), and in which group	N/A
	27c	How missing data will be handled in the analysis	N/A
	27d	Methods for any additional analyses (for example, subgroup and sensitivity analyses)	Explain whether a subgroup analysis considers dog characteristics (e.g., size)
*Methods: Monitoring*			
Data monitoring committee	28a	Composition of data monitoring committee (DMC); summary of its role and reporting structure; statement of whether it is independent from the sponsor and funder; conflicts of interest and reference to where further details about its charter can be found, if not in the protocol. Alternatively, an explanation of why a DMC is not needed	Summary of the role of the DMC or other relevant committees in relation to monitoring dog welfare
	28b	Explanation of any interim analyses and stopping guidelines, including who will have access to these interim results and make the final decision to terminate the trial	Justification, and description, of whether interim analyses and stopping guidelines consider dog welfare
Trial monitoring	29	Frequency and procedures for monitoring trial conduct. If there is no monitoring, give explanation	Plans for collecting, assessing, reporting and managing harms or unintended effects for the dog(s)
*Ethics*			
Research ethics approval	30	Plans for seeking research ethics committee/institutional review board approval	N/A
Protocol amendments	31	Plans for communicating important protocol modifications to relevant parties	N/A
Consent or assent	32a	Who will obtain informed consent or assent from potential trial participants or authorized proxies, and how	N/A
	32b	Additional consent provisions for collection and use of participant data and biological specimens in ancillary studies, if applicable	N/A
Confidentiality	33	How personal information about potential and enrolled participants will be collected, shared, and maintained in order to protect confidentiality before, during, and after the trial	N/A
Ancillary and post-trial care	34	Provisions, if any, for ancillary and post-trial care, and for compensation to those who suffer harm from trial participation	Description for post-session and/or post-intervention care in relation to dog wellbeing where applicable
			Provisions for supporting the end of interactions between the dog and participant (post-session and/or post-intervention)

**Table 3:** Checklist for reporting of DAIs: extension of the CONSORT 2025 statement

Section/topic	No	CONSORT 2025 checklist item	Extension for DAI RCTs
*Title and abstract*			
Title and structured abstract	1a	Identification as a randomised trial	The species of animal involved (i.e., dog)
			Type of DAI (e.g., therapy, activity, education)
	1b	Structured summary of trial design, methods, results, and conclusions	N/A
*Open science*			
Trial registration	2	Name of trial registry, identifying number (with URL) and date of registration	N/A
Protocol and statistical analysis plan	3	Where the trial protocol and statistical analysis plan can be accessed	N/A
Data sharing	4	Where and how the individual de-identified participant data (including data dictionary), statistical code and any other materials can be accessed	N/A
Funding and conflicts of interest	5a	Sources of funding and other support (e.g., supply of drugs), and role of funders in the design, conduct, analysis, and reporting of the trial	N/A
	5b	Financial and other conflicts of interest of the manuscript authors	Name, accreditation status, non-/for-profit status of any animal-assisted intervention organisations involved in intervention (where applicable)
*Introduction*			
Background and rationale	6	Scientific background and rationale	Scientific background and rationale for including a DAI
			Description of proposed mechanism(s), model(s), or theories describing the potential impact of the DAI, if applicable
Objectives	7	Specific objectives related to benefits and harms	Specific objectives or hypotheses related to the DAI
*Methods*			
Patient and public involvement	8	Details of patient or public involvement in the design, conduct and reporting of the trial	N/A
Trial design	9	Description of trial design including type of trial (e.g., parallel group, crossover), allocation ratio, and framework (e.g., superiority, equivalence, non-inferiority, exploratory)	N/A
Changes to trial protocol	10	Important changes to the trial after it commenced including any outcomes or analyses that were not prespecified, with reason	N/A
Trial setting	11	Settings (e.g., community, hospital) and locations (e.g., countries, sites) where the trial was conducted	Description of how considerations related to dog welfare are incorporated in the study context and settings
			Description of the characteristics of study settings and the DAI that may affect dogs (e.g., room size, noises, smells, lighting)
			Description of how the suitability of the dog for the DAI in the given study context and environment is ensured
Eligibility criteria	12a	Eligibility criteria for participants	Eligibility criteria relevant to engaging/interacting with dog, if any (e.g., dog phobia, allergies, history of dog abuse)
	12b	If applicable, eligibility criteria for sites and for individuals delivering the interventions (e.g., surgeons, physiotherapists)	Selection criteria for the dog(s) involved in the intervention and justification for these
			Description of any criteria for sites to ensure dog welfare requirements can be met
			Description of relevant training and education undertaken by all parties that is pertinent to their role in the intervention (e.g., participant vs. dog handler). This may include training on dog welfare, safety and intervention-specific content
Intervention and comparator	13	Intervention and comparator with sufficient details to allow replication. If relevant, where additional materials describing the intervention and comparator (e.g., intervention manual) can be accessed	Roles and responsibilities of individual(s) responsible for animal welfare
			State which DAI guidelines/code of practice are used if appropriate
			Description of DAI goal and content
			Description of the proposed duration and frequency of DAI including, where possible, justification
			Description of tasks and roles of each individual in the DAI team (i.e., dog, dog handler, other trained professional) including details of participant-dog interactions
			Where possible, consideration of suitability/tailoring of DAI under equality, diversity and inclusion aspects (e.g., severity of illness)
			Rationale for and description of the comparator group
Outcomes	14	Prespecified primary and secondary outcomes, including the specific measurement variable (e.g., systolic blood pressure), analysis metric (e.g., change from baseline, final value, time to event), method of aggregation (e.g., median, proportion), and time point for each outcome	Outcome measures relevant to the proposed pathway of action of the DAI
			Where applicable, justify the use of unblinded outcome measures in relation to the DAI
Harms	15	How harms were defined and assessed (e.g., systematically, non-systematically)	Observations/measures relevant to the impact of the intervention on the dog(s)
Sample size	16a	How sample size was determined	Description of capacity of dog-handler teams required to safely and effectively deliver the intervention
	16b	Explanation of any interim analyses and stopping guidelines	Justification, and description, of whether interim analyses and stopping guidelines consider dog welfare
Sequence generation	17a	Who generated the random allocation sequence, and the method used	N/A
	17b	Type of randomisation and details of any restriction (e.g., stratification, blocking, and block size)	Description of whether concomitant interactions with dogs (e.g., interactions with pet/other dogs) is included as a stratification variable
Allocation concealment mechanism	18	Mechanism used to implement the random allocation sequence (e.g., central computer/telephone; sequentially numbered, opaque, sealed containers), describing any steps to conceal the sequence until interventions were assigned	N/A
Implementation	19	Whether the personnel who enrolled and those who assigned participants to the interventions had access to the random allocation sequence	If possible, describe the process of matching dog-handler teams (dog & handler) to participants, including the individual/team responsible for this process, where relevant
Blinding	20a	Who was blinded after assignment to interventions (e.g., participants, care providers, outcome assessors, data analysts)	Description and justification of whether trained professional(s) and dog handler(s) are blinded to the outcomes and how
	20b	If blinded, how blinding was achieved and description of the similarity of interventions	N/A
Statistical methods	21a	Statistical methods used to compare groups for primary and secondary outcomes, including harms	N/A
	21b	Definition of who is included in each analysis (e.g., all randomised participants), and in which group	N/A
	21c	How missing data were handled in the analysis	N/A
	21d	Methods for any additional analyses (e.g., subgroup and sensitivity analyses), distinguishing prespecified from post hoc	N/A
*Results*			
Participant flow, including flow diagram	22a	For each group, the numbers of participants who were randomly assigned, received intended intervention, and were analysed for the primary outcome	Time schedule and completion rates of any required participant-dog training/familiarity sessions prior to commencement of the intervention
	22b	For each group, losses and exclusions after randomisation, together with reasons	Participant losses/exclusions for reasons relating to interacting with a dog
			Losses/exclusions of the dog(s)/dog handler(s) with reasons why
Recruitment	23a	Dates defining the periods of recruitment and follow-up for outcomes of benefits and harms	N/A
	23b	If relevant, why the trial ended or was stopped	N/A
Intervention and comparator delivery	24a	Intervention and comparator as they were actually administered (e.g., where appropriate, who delivered the intervention/comparator, whether participants adhered, whether they were delivered as intended (fidelity))	N/A
	24b	Concomitant care received during the trial for each group	N/A
Baseline data	25	A table showing baseline demographic and clinical characteristics for each group	Baseline data indicating concomitant interactions with dogs (e.g., interactions with pets/other dogs) if relevant
			Description of the number and characteristics of the dog(s) and their handler(s) involved
Numbers analysed, outcomes, and estimation	26	For each primary and secondary outcome, by group: • the number of participants included in the analysis • the number of participants with available data at the outcome time point • result for each group, and the estimated effect size and its precision (such as 95% confidence interval) • for binary outcomes, presentation of both absolute and relative effect size	N/A
Harms	27	All important harms or unintended effects in each group (for specific guidance, see CONSORT for harms)	Harms or unintended effects associated with the DAI, for the dog, and how these were assessed
Ancillary analyses	28	Any other analyses performed, including subgroup and sensitivity analyses, distinguishing prespecified from post hoc	Explain whether a subgroup analysis considers dog characteristics (e.g., size)
*Discussion*			
Interpretation	29	Interpretation consistent with results, balancing benefits and harms, and considering other relevant evidence	N/A
Limitations	30	Trial limitations, addressing sources of potential bias, imprecision, generalisability, and, if relevant, multiplicity of analyses	N/A

#### Administrative/title and structured abstract

SPIRIT 1a: Title stating the trial design, population, and interventions, with identification as a protocol.

CONSORT 1a: Identification as a randomised trial.

- ➢ Extension for DAI trials: The species of animal involved (i.e., dog).

Alerts the reader to the type of animal involved and the relevancy of the paper.

SPIRIT 1a: Title stating the trial design, population, and interventions, with identification as a protocol.

CONSORT 1a: Identification as a randomised trial.

- ➢ Extension for DAI trials: Type of animal-assisted intervention (e.g., therapy, activity, or education).

The nature of interventions with a dog can vary considerably (see Table 1). Clearly identifying the type of intervention promotes clarity and relevance for readers.

SPIRIT 3d: Composition, roles, and responsibilities of the coordinating site, steering committee, endpoint adjudication committee, data management team, and other individuals or groups overseeing the trial, if applicable.

CONSORT 13: Intervention and comparator with sufficient details to allow replication. If relevant, where additional materials describing the intervention and comparator (e.g., intervention manual) can be accessed.

- ➢ Extension for DAI trials: Roles and responsibilities of individual(s) responsible for dog welfare.
- ➢ Extension for DAI trials: State which DAI guidelines/code of practice are used if appropriate.

As well as promoting adherence to welfare/ethical standard, reporting this helps to document any potentially competing interests.

#### Open science

SPIRIT 3d: Composition, roles, and responsibilities of the coordinating site, steering committee, endpoint adjudication committee, data management team, and other individuals or groups overseeing the trial, if applicable.

CONSORT 5b: Financial and other conflicts of the manuscript authors.

- ➢ Extension for DAI trials: Name, accreditation status, non-/for-profit status of any AAI organisations involved in intervention (where applicable).

Important for assessing conflicts of interest and maintain transparency relating to the involvement of the dog.

#### Introduction

SPIRIT 9a: Scientific background and rationale, including summary of relevant studies (published and unpublished) examining benefits and harms for each intervention.

CONSORT 6: Scientific background and rationale.

- ➢ Extension for DAI trials: Scientific background and rationale for including a DAI.
- ➢ Extension for DAI trials: Description of proposed mechanism(s), model(s) or theories describing the potential impact of the DAI if applicable.

DAI are often viewed as having low theoretical credibility. Presenting the scientific background ensures the intervention is grounded in sound theory, aligns with the research objectives, and promotes an understanding of how the research aids scientific enquiry.

SPIRIT 10: Specific objectives related to benefits and harms.

CONSORT 7: Specific objectives related to benefits and harms.

- ➢ Extension for DAI trials: Specific objectives or hypotheses related to the DAI.

Helps the reader to understand why (e.g., for reducing participant arousal) and how a DAI (e.g., as a stand-alone intervention or an adjunct to an existing treatment) was used.

#### Methods

SPIRIT 13: Settings (for example, community, hospital) and locations (for example, countries, sites) where the trial will be conducted.

CONSORT 11: Settings (e.g., community, hospital) and locations (e.g., countries, sites) where the trial was conducted.

- ➢ Extension for DAI trials: Description of how considerations related to dog welfare are incorporated in the study context and settings.
- ➢ Extension for DAI trials: Description of the characteristics of study settings and the DAI which may affect the dog(s) (e.g., room size, noises, smells, lighting).
- ➢ Extension for DAI trials: Description of how the suitability of the dog for the DAI in the given study context and environment is ensured.

These three extensions help ensure the suitability and welfare of the dog are considered in relation to the study settings and locations.

SPIRIT 14a: Eligibility criteria for participants.

CONSORT 12a: Eligibility criteria for participants.

- ➢ Extension for DAI trials: Eligibility criteria relevant to engaging/interacting with the dog, if any (e.g., dog phobia, allergies, history of dog abuse).

This helps to ensure participant safety and dog welfare.

SPIRIT 14b: If applicable, eligibility criteria for sites and for individuals who will deliver the interventions (for example, surgeons, physiotherapists).

CONSORT 12b: If applicable, eligibility criteria for sites and for individuals delivering the interventions (e.g., surgeons, physiotherapists).

- ➢ Extension for DAI trials: Description of any criteria for sites to ensure dog welfare requirements can be met.
- ➢ Extension for DAI trials: Selection criteria for the dog(s) involved in the intervention and justification for these.
- ➢ Extension for DAI trials: Description of relevant training and education undertaken by all parties that is pertinent to their role in the intervention (e.g., participant vs dog handler). This may include training on dog welfare, safety, and intervention specific content.

These extensions, three which apply to SPIRIT and two which apply to CONSORT, encourage the reporting of criteria which ensure the setting and the individuals involved in delivering the intervention are suitable for the type/level of dog contact required.

SPIRIT 15a: Intervention and comparator with sufficient details to allow replication including how, when, and by whom they will be administered. If relevant, where additional materials describing the intervention and comparator (for example, intervention manual) can be accessed.

CONSORT 13: Intervention and comparator with sufficient details to allow replication. If relevant, where additional materials describing the intervention and comparator (e.g., intervention manual) can be accessed.

- ➢ Extension for DAI trials: Description of DAI goal and content.
- ➢ Extension for DAI trials: Description of the proposed duration and frequency of DAI including, where possible, justification.
- ➢ Extension for DAI trials: Description of tasks and roles of each individual in the DAI team (i.e., dog, dog handler, other trained professional) including details on participant-dog interactions.
- ➢ Extension for DAI trials: Where possible, consideration of suitability/tailoring of DAI under EDI aspects (e.g., severity of illness).
- ➢ Extension for DAI trials: Rationale for and description of the comparator group.

Provides important detail surrounding the role of the dog in the intervention to enable replication and evaluate potential safety and welfare concerns.

SPIRIT 15b: Criteria for discontinuing or modifying allocated intervention/comparator for a trial participant (for example, drug dose change in response to harms, participant request, or improving/worsening disease).

- ➢ Extension for DAI trials: Criteria and/or processes for discontinuing or modifying the intervention based on dog and/or handler responses.

Ensures there is a clear process for protecting all involved (e.g., dog, participant, handler) based upon individual responses to the intervention.

SPIRIT 15d: Concomitant care that is permitted or prohibited during the trial.

- ➢ Extension for DAI trials: Description of whether and how potential concomitant human-dog interactions (e.g., with pet dogs) are controlled for.

Participants experience with, and preferences for, dog contact may influence the outcome of the DAI.

SPIRIT 16: Primary and secondary outcomes, including the specific measurement variable (for example, systolic blood pressure), analysis metric (for example, change from baseline, final value, time to event), method of aggregation (for example, median, proportion), and time point for each outcome.

CONSORT 14: Prespecified primary and secondary outcomes, including the specific measurement variable (e.g., systolic blood pressure), analysis metric (e.g., change from baseline, final value, time to event), method of aggregation (e.g., median, proportion), and time point for each outcome.

- ➢ Extension for DAI trials: Outcome measures relevant to the proposed pathway of action of the DAI.
- ➢ Extension for DAI trials: Where applicable, justify the use of unblinded outcome measures in relation to the DAI.

Ensures the outcome measures are directly relevant to the nature of the DAI.

SPIRIT 17: How harms are defined and assessed (for example, systematically, non-systematically).

CONSORT 15: How harms were defined and assessed (e.g. systematically, non-systematically).

- ➢ Extension for DAI trials: Observations/measures relevant to the impact of the intervention on the dog(s).

Ensures that assessment of harms consider the outcome(s) for the dog.

SPIRIT 18: Time schedule of enrolment, interventions (including any run-ins and washouts), assessments, and visits for participants. A schematic diagram is highly recommended.

CONSORT 22a: For each group, the numbers of participants who were randomly assigned, received intended intervention, and were analysed for the primary outcome.

- ➢ Extension for DAI trials—SPIRIT: Time schedule of any required participant-dog training/familiarity sessions prior to commencement of the intervention.
- ➢ Extension for DAI trials—CONSORT: Time schedule and completion rates of any required participant-dog training/familiarity sessions prior to commencement of the intervention.

Familiarity sessions are important to support positive interactions for all parties (i.e., participant and dog).

SPIRIT 19: How sample size was determined, including all assumptions supporting the sample size calculation.

CONSORT 16a: How sample size was determined.

- ➢ Extension for DAI trials: Description of capacity of dog-handler teams required to safely and effectively deliver the intervention.

The number of dog-handler teams (i.e., dogs and handlers) need to be estimated reliably to ensure there is capacity to deliver the intervention without compromising dog welfare or integrity of the intervention.

SPIRIT 28b: Explanation of any interim analyses and stopping guidelines, including who will have access to these interim results and make the final decision to terminate the trial.

CONSORT 16b: Explanation of any interim analyses and stopping guidelines.

- ➢ Extension for DAI trials: Justification, and description, of whether interim analyses and stopping guidelines consider dog welfare.

Ensures the trial can be modified or stopped if it has a negative impact on the dogs involved.

SPIRIT 21b: Type of randomization (simple or restricted) and details of any factors for stratification. To reduce predictability of a random sequence, other details of any planned restriction (for example, blocking) should be provided in a separate document that is unavailable to those who enrol participants or assign interventions.

CONSORT 17b: Type of randomisation and details of any restriction (e.g. stratification, blocking and block size).

- ➢ Extension for DAI trials: Description of whether concomitant interactions with dogs (e.g., interactions with pet/other dogs) is included as a stratification variable.

Stratification ensures that groups (e.g., intervention and control groups) are balanced in terms of participants’ pre-existing dog interactions, supporting the reliability and generalisability of the results.

SPIRIT 21a: Who will generate the random allocation sequence, and the method used.

CONSORT 19: Whether the personnel who enrolled and those who assigned participants to the interventions had access to the random allocation sequence.

- ➢ Extension for DAI trials: If possible, describe the process of matching dog-handler teams (dog & handler) to participants, including the individual/team responsible for this process, where relevant.

A well described matching process helps to support positive dog-participant interactions.

SPIRIT 24a: Who will be blinded after assignment to interventions (for example, participants, care providers, outcome assessors, data analysts).

CONSORT 20a: Who was blinded after assignment to interventions (e.g., participants, care providers, outcome assessors, data analysts).

- ➢ Extension for DAI trials: Description and justification of whether trained professional(s) and dog handler(s) are blinded to the outcomes and how.

Interaction between the dog, handler, and participants is central to a DAI. Awareness of expected outcomes might unconsciously alter how the handler or professional facilitates these interactions. However, blinding is not always possible.

SPIRIT 27b: Methods for any additional analyses (e.g., subgroup and sensitivity analyses).

CONSORT 28: Any other analyses performed, including subgroup and sensitivity analyses, distinguishing prespecified from post hoc.

- ➢ Extension for DAI trials: Explain whether a subgroup analysis considers dog characteristics (e.g., size).

Characteristics associated with the dogs involved in a DAI are challenging to standardize, subgroup analyses can help identify the potential impact of this.

SPIRIT 28a: Composition of data monitoring committee (DMC); summary of its role and reporting structure; statement of whether it is independent from the sponsor and funder; conflicts of interest and reference to where further details about its charter can be found, if not in the protocol. Alternatively, an explanation of why a DMC is not needed.

- ➢ Extension for DAI trials: Summary of the role of the DMC or other relevant committee in relation to monitoring dog welfare.<div class="NodiCopyInline">**Extension for DAI trials:** Summary of the role of the DMC or other relevant committee in relation to monitoring dog welfare.</div>

Independent, appropriately trained experts are important to ensure dog welfare is considered at all times during the trial.

SPIRIT 29: Frequency and procedures for monitoring trial conduct. If there is no monitoring, give explanation.

CONSORT 27: All important harms or unintended effects in each group.

- ➢ Extension for DAI trials – SPIRIT: Plans for collecting, assessing, reporting and managing harms or unintended effects for the dog(s).
- ➢ Extension for DAI trials—CONSORT: Harms or unintended effects associated with the DAI, for the dog, and how these were assessed.

Reporting the harms/unintended effects of the intervention for the dog supports ethical management of the dog throughout the trial.

CONSORT 22b: For each group, losses and exclusions after randomisation, together with reasons.

- ➢ Extension for DAI trials: Participant losses/exclusions for reasons relating to interacting with a dog.
- ➢ Extension for DAI trials: Losses/exclusions of the dog(s)/dog handler(s) with reasons why.

Reporting losses/exclusions relating specifically to the dog is important to identify potential barriers to implementation in practice and implications for reliability and generalisability of the results.

CONSORT 25: A table showing baseline demographic and clinical characteristics for each group.

- ➢ Extension for DAI trials: Baseline data indicating concomitant interactions with dogs (e.g., interactions with pets/other dogs) if relevant.
- ➢ Extension for DAI trials: Description of the number and characteristics of the dog(s) and their handler(s) involved.

Variations in dog-related factors (e.g., age, size, breed, training level) and handler-related factors (e.g., gender, ethnicity, length of experience) may influence intervention outcomes and generalisability of the results.

SPIRIT 34: Provisions, if any, for ancillary and post-trial care, and for compensation to those who suffer harm from trial participation.

- ➢ Extension for DAI trials: Description for post-session and/or post-intervention care in relation to dog wellbeing where applicable.
- ➢ Extension for DAI trials: Provisions for supporting and managing the end of interactions between the dog and participant (post-session and/or post-intervention).

Reporting this ensures dog and participant wellbeing are appropriately considered outside of the context of the intervention.

## Discussion

This extension to the SPIRIT 2025 and CONSORT 2025 Statement provides guidance for reporting on the design and results of DAIs. The extension checklist represents the minimum essential requirements for reporting trials in this field. This should not be considered an exhaustive list; additional reporting items may be necessary, depending on the design of the trial.

Together, these guidelines provide a comprehensive framework for reporting DAI trials, from protocol to publication of the results. It is hoped that uptake of these guidelines will improve the quality and scientific rigour of DAI research, helping to mitigate against many instances of poor reporting identified in a recent systematic review [22]. Using a mixed-methods approach, incorporating a substantial Delphi survey sample, and adhering to procedures recommended by EQUATOR and applied by other research teams developing extension documents [57, 58]. However, additional concepts relevant to DAI trials may warrant inclusion in study protocols and manuscripts. Indeed, the systematic review and search of study protocols may not have enabled us to identify all shortcomings with reporting in this field. Whilst we provided Delphi survey respondents with the opportunity to suggest additional items to mitigate this, we recommend viewing these extensions as a minimum set of reporting items, consistent with the objectives of the CONSORT and SPIRIT statements. While our Delphi sample included a diverse range of DAI experts, from researchers to animal handlers, fewer than half had experience with DAI RCTs. Nonetheless, the sample demonstrated considerable expertise, with an average of 11 years’ experience in DAI and seven academic publications. In addition, the majority of participants were White, from Europe or North America, and were researchers or academics. This represents the dominance of DAI in high as opposed to low-income countries, and challenges with conducting Delphi surveys with non-academic participants. As DAIs continue to grow, more global perspectives, including from those conducting research in low resource settings, may be valuable to ensuring the proposed extension remains a globally relevant framework. However, to ensure the perspectives of animal handlers and trainers informed the proposed extension items, we worked closely with a UK-based DAI charity handler and other independent professionals in our PPI) group. Through guided survey piloting and consultation sessions, these DAI experts provided valuable feedback that grounded the extension items in real-world practice and prioritised the wellbeing of animals, handlers, and all parties involved in DAI research. A further point to note is that we did not include service users in our Delphi survey, due to challenges with engaging public members with a relatively complex methodological process Whilst PPI is not commonly considered in the development of reporting guidelines, patients often do their own information seeking with regards to healthcare options [59–61] and therefore developing guidelines which promote the readability of this information to patients could be important. Future research should include a PPI evaluation of the guidelines developed here. The review, which informed the original development of the draft SPIRIT and CONSORT items, focused on RCTs to align with the trial-reporting focus of SPIRIT and CONSORT. However, dog-assisted intervention research also includes non-randomised designs (e.g., cohort or quasi-experimental studies), which may contain additional methodological or reporting considerations that were not captured in our review. In addition, the review was restricted to studies involving mental health and neurodevelopmental conditions, reflecting the current concentration of DAI trials in these populations. DAI research is also emerging in other clinical areas, including ageing and dementia populations such as those with Alzheimer's disease, and future work may wish to consider whether evidence from these domains could further inform reporting guidance.

## Conclusion

This consensus statement describing an extension of the SPIRIT 2025 and CONSORT 2025 Statements provides specific guidance for the reporting of DAI trial protocols and manuscripts. This guidance should help provide greater transparency and completeness in the reporting of DAI trials.

## Supplementary Information

Supplementary Material 1.

Supplementary Material 2.

Supplementary Material 3.

## Data Availability

The datasets used and/or analysed during the current study are available from the corresponding author on reasonable request.

## Abbreviations

AAIs: Animal-Assisted Interventions
DAI: Dog-assisted Intervention
CONSORT: Consolidated Standard of Reporting Trials
RCT: Randomised Controlled Trial
SPIRIT: Standard Protocol Items: Recommendations for Interventional Trials
