# Supplementary material for: Reporting of dog-assisted intervention trials: extension of the SPIRIT 2025 and CONSORT 2025 statement
Source: BMC Med Res Methodol. 2026 Apr 20;26:113. doi: 10.1186/s12874-026-02848-7 (PMC13173953; doi:10.1186/s12874-026-02848-7)
Supplement: Supplementary file 1 — Supplementary Material 1. [file 12874_2026_2848_MOESM1_ESM.docx]

**Supplementary Material 1. Participant characteristics in Delphi survey rounds one, two and three.**

| **Participant Characteristics** | | | |
| --- | --- | --- | --- |
|  | **Round 1 (N=83)** | **Round 2 (N=74)** | **Round 3 (N=71)** |
|  | **N (%)** | | |
| **Age** |  |  |  |
| 26-34 years | 10 (12%) | 10 (14%) | 9 (13%) |
| 35-44 years | 24 (29%) | 22 (30%) | 21 (30%) |
| 45-55 years | 25 (30%) | 21 (28%) | 20 (28%) |
| 56-65 years | 19 (23%) | 16 (22%) | 16 (23%) |
| > 66 years | 4 (5%) | 4 (5%) | 4 (6%) |
| Prefer not to say | 1 (1%) | 1 (1%) | 1 (1%) |
| **Gender** |  |  |  |
| Female | 62 (75%) | 54 (73%) | 51 (72%) |
| Male | 19 (23%) | 18 (24%) | 18 (25%) |
| Non-binary | 1 (1%) | 1 (1%) | 1 (1%) |
| Prefer not to say | 1 (1%) | 1 (1%) | 1 (1%) |
| **Ethnicity** |  |  |  |
| White | 76 (92%) | 67 (91%) | 65 (92%) |
| Asian | 2 (2%) | 2 (3%) | 2 (3%) |
| Black | 1 (1%) | 1 (1%) | 1 (1%) |
| Other | 2 (2%) | 2 (3%) | 1 (1%) |
| Prefer not to say | 2 (2%) | 2 (3%) | 2 (3%) |
| **Continent** |  |  |  |
| Europe | 53 (64%) | 46 (62%) | 44 (62%) |
| Asia | 2 (2%) | 2 (3%) | 2 (3%) |
| North America | 21 (25%) | 19 (26%) | 18 (25%) |
| Oceania | 7 (8%) | 7 (9%) | 7 (10%) |
| **Main role** |  |  |  |
| Animal handler/AAI provider | 9 (11%) | 8 (11%) | 8 (11%) |
| Researcher/Academic | 56 (67%) | 52 (70%) | 50 (70%) |
| Therapist/Counsellor/Clinician | 18 (22%) | 14 (19%) | 13 (18%) |
| **Involvement in AAI Randomised-Controlled Trials** | | | |
| Yes | 32 (39%) | 28 (38%) | 28 (39%) |
| No | 51 (61%) | 46 (62%) | 43 (61%) |
| **Years of experience in AAI** |  |  |  |
| Average *(M)* | 11.03 | 11.06 | 11.15 |
| Min *(N)* | 0 | 0 | 0 |
| Max *(N)* | 50 | 50 | 50 |
| **AAI peer-reviewed papers** |  |  |  |
| Average *(M)* | 6.96 | 7.18 | 7.31 |
| Min *(N)* | 0 | 0 | 0 |
| Max *(N)* | 76 | 76 | 76 |

*AAI= Animal-Assisted Intervention*
