# Supplementary material for: Reporting of dog-assisted intervention trials: extension of the SPIRIT 2025 and CONSORT 2025 statement
Source: BMC Med Res Methodol. 2026 Apr 20;26:113. doi: 10.1186/s12874-026-02848-7 (PMC13173953; doi:10.1186/s12874-026-02848-7)
Supplement: Supplementary file 2 — Supplementary Material 2. [file 12874_2026_2848_MOESM2_ESM.docx]

**Supplementary Material 2. Delphi Survey Round 2 item wording revisions**

| **Original DAI Item** | **Updated wording** | **Clarification added** |
| --- | --- | --- |
| **Introduction** |  |  |
| ﻿﻿  CONSORT: Scientific background and rationale for including a DAI | No | We use the term DAI to refer to the dog, the handler and/or a trained professional. |
| SPIRIT: Description of proposed mechanism(s) of action and/or logic model describing the potential impact of the DAI | SPIRIT: Description of proposed mechanism(s), model(s) or theories describing the potential impact of the DAI if applicable | No |
| CONSORT: Description of proposed mechanism(s) of action and/or logic model describing the potential impact of the DAI | CONSORT: Description of proposed mechanism(s), model(s) or theories describing the potential impact of the DAI if applicable | No |
| SPIRIT: Specific objectives/hypotheses in relation to the DAI impact | SPIRIT: Specific objectives and hypotheses in relation to the DAI impact | No |
| CONSORT: Specific objectives/hypotheses in relation to the DAI impact | CONSORT: Specific objectives and hypotheses in relation to the DAI impact | No |
| **Methods** |  |  |
| SPIRIT: Justify the suitability of the dog in relation to the environment | SPIRIT: Justify the suitability of the dog in relation to the social and physical environment | Social (e.g., patient group) and physical (e.g. noise levels). |
| CONSORT: Describe the suitability of the dog in relation to the environment | CONSORT: Describe the suitability of the dog in relation to the social and physical environment | Social (e.g., patient group) and physical (e.g. noise levels). |
| SPIRIT: Describe the on- and off-site requirements in relation to integrating the DAI into the trial setting | No | These requirements may include training, a risk assessment, insurance etc. |
| CONSORT: Describe the on- and off-site requirements in relation to integrating the DAI into the trial setting | No | These requirements may include training, a risk assessment, insurance etc. |
| SPIRIT: Selection criteria for the dog(s) involved in the intervention and justification for these | No | Inclusion/exclusion criteria for the dogs to be included here. |
| CONSORT: Selection criteria for the dog(s) involved in the intervention and justification for these | No | Inclusion/exclusion criteria for the dogs to be included here. |
| SPIRIT: Eligibility criteria relevant to interacting with the dog | No | Participant eligibility relevant to taking part in an intervention involving a dog (for example, allergies). |
| CONSORT: Eligibility criteria relevant to interacting with the dog | No | Participant eligibility relevant to taking part in an intervention involving a dog (for example, allergies) |
| SPIRIT: Eligibility criteria for therapist(s)/handler(s) | SPIRIT: Eligibility criteria for trained professional(s) and/or handler(s) | Trained professionals may include clinicians, nurses, teachers etc. |
| CONSORT: Eligibility criteria for therapist(s)/handler(s) | CONSORT: Eligibility criteria for trained professional(s) and/or handler(s) | Trained professionals may include clinicians, nurses, teachers etc. |
| SPIRIT: Description of intervention-specific training completed by dog-handler teams | SPIRIT: Description of intervention-specific training completed by dog and dog-handler teams | No |
| CONSORT: Description of intervention-specific training completed by dog-handler teams | CONSORT: Description of intervention-specific training completed by dog and dog-handler teams | No |
| SPIRIT: Description of tasks and roles of each individual in the DAI team (i.e., dog, dog handler, therapist) including details on participant-dog interactions | SPIRIT: Description of tasks and roles of each individual in the DAI team (i.e., dog, dog handler, other trained professional) including details on participant-dog interactions | No |
| CONSORT: Description of tasks and roles of each individual in the DAI team (i.e., dog, dog handler, therapist) including details on participant-dog interactions | CONSORT: Description of tasks and roles of each individual in the DAI team (i.e., dog, dog handler, other trained professional) including details on participant-dog interactions | No |
| SPIRIT: Criteria and processes for discontinuing or modifying the intervention based on observed, handler and participant responses | SPIRIT: Criteria and processes for discontinuing or modifying the intervention based on dog, handler and participant responses | No |
| SPIRIT: Outcome measures relevant to the proposed pathway of action of the DAI | No | Where relevant to the study. |
| CONSORT: Outcome measures relevant to the proposed pathway of action of the DAI | No | Where relevant to the study. |
| SPIRIT: Description and justification of whether therapist(s) and dog handler(s) will be blinded to the outcomes | SPIRIT: Description and justification of whether trained professional(s) and dog handler(s) will be blinded to the outcomes | Trained professionals may include clinicians, nurses, therapists etc. |
| CONSORT: Description and justification of whether therapist(s) and dog handler(s) were blinded to the outcomes | CONSORT: Description and justification of whether trained professional(s) and dog handler(s) were blinded to the outcomes | Trained professionals may include clinicians, nurses, therapists etc. |
| **Results** |  |  |
| SPIRIT: Describe the process of matching the dog handler teams (dog & handler) with individual participants | SPIRIT: Describe the process of matching the dog handler teams (dog & handler) with participants | If appropriate, how were dog handler teams assigned to individual participants, or the participant group based upon their suitability for each other. |
| CONSORT: Describe the process of matching the dog handler teams (dog & handler) with individual participants | CONSORT: Describe the process of matching the dog handler teams (dog & handler) with participants | If appropriate, how were dog handler teams assigned to individual participants, or the participant group based upon their suitability for each other. |
| CONSORT: Who will select the dog handler teams (dog & handler) for the DAI | CONSORT: Who selected the dog handler teams (dog & handler) for the DAI | No |
