# Supplementary material for: Reporting of dog-assisted intervention trials: extension of the SPIRIT 2025 and CONSORT 2025 statement
Source: BMC Med Res Methodol. 2026 Apr 20;26:113. doi: 10.1186/s12874-026-02848-7 (PMC13173953; doi:10.1186/s12874-026-02848-7)
Supplement: Supplementary file 3 — Supplementary Material 3. [file 12874_2026_2848_MOESM3_ESM.docx]

**Supplementary Material 3. Final Dog-Assisted Intervention (DAI) item wording following consensus workshop review**

| **Checklist** | **Item #** | **Original Item** | **DAI Extension** | **Workshop/Team Edits** | **Final Wording** |
| --- | --- | --- | --- | --- | --- |
| SPIRIT | 1a | Title stating the trial design, population, and interventions, with identification as a protocol | The species of animal involved (i.e., dog) | N/A | The species of animal involved (i.e., dog) |
| CONSORT | 1a | Identification as a randomised trial | The species of animal involved (i.e., dog) | N/A | The species of animal involved (i.e., dog) |
| SPIRIT | 1a | Title stating the trial design, population, and interventions, with identification as a protocol | Type of animal-assisted intervention (e.g., therapy, activity, or education) | Change animal to dog | Type of dog-assisted intervention (e.g., therapy, activity, or education) |
| CONSORT | 1a | Identification as a randomised trial | Type of animal-assisted intervention (e.g., therapy, activity, or education) | Change animal to dog | Type of dog-assisted intervention (e.g., therapy, activity, or education) |
| SPIRIT | 3d | Composition, roles, and responsibilities of the coordinating site, steering committee, endpoint adjudication committee, data management team, and other individuals or groups overseeing the trial, if applicable | Roles and responsibilities of individual(s) responsible for dog welfare | This should also be a CONSORT item | Roles and responsibilities of individual(s) responsible for dog welfare |
| CONSORT | 13 | The interventions for each group with sufficient details to allow replication, including how and when they were actually administered | Not included in the Delphi survey | This should also be a CONSORT item | Roles and responsibilities of individual(s) responsible for dog welfare |
| SPIRIT | 3d | Composition, roles, and responsibilities of the coordinating site, steering committee, endpoint adjudication committee, data management team, and other individuals or groups overseeing the trial, if applicable | State which AAI guidelines/code of practice are used if appropriate | AAI should be made dog specific - change to DAI | State which DAI guidelines/code of practice are used if appropriate |
| CONSORT | 13 | The interventions for each group with sufficient details to allow replication, including how and when they were actually administered | State which AAI guidelines/code of practice were used if appropriate | AAI should be made dog specific - change to DAI | State which DAI guidelines/code of practice are used if appropriate |
| SPIRIT | 3d | Composition, roles, and responsibilities of the coordinating site, steering committee, endpoint adjudication committee, data management team, and other individuals or groups overseeing the trial, if applicable | Name, accreditation status, non-/for-profit status of any AAI organisations involved in intervention. | May not always be applicable - note this | Name, accreditation status, non-/for-profit status of any AAI organisations involved in intervention (where applicable) |
| SPIRIT | 7a | Sources of funding and other support (for example, supply of drugs) | Financial and other competing interests for the dog handler/facilitator/ organisation | Remove this item due to lack of specificity to dog assisted interventions and similar to a previous item | N/A |
| CONSORT | 5b | Financial and other conflicts of interest of the manuscript author | Name, accreditation status, non-/for-profit status of any AAI organisations involved in intervention. | May not always be applicable - note this | Name, accreditation status, non-/for-profit status of any AAI organisations involved in intervention (where applicable) |
| SPIRIT | 9a | Scientific background and rationale, including summary of relevant studies (published and unpublished) examining benefits and harms for each intervention | Scientific background and rationale for including a DAI | N/A | Scientific background and rationale for including a DAI |
| CONSORT | 6 | Scientific background and rationale | Scientific background and rationale for including a DAI | N/A | Scientific background and rationale for including a DAI |
| SPIRIT | 9a | Scientific background and rationale, including summary of relevant studies (published and unpublished) examining benefits and harms for each intervention | Description of proposed mechanism(s), model(s) or theories describing the potential impact of the DAI if applicable | N/A | Description of proposed mechanism(s), model(s) or theories describing the potential impact of the DAI if applicable |
| CONSORT | 6 | Scientific background and rationale | Description of proposed mechanism(s), model(s) or theories describing the potential impact of the DAI if applicable | N/A | Description of proposed mechanism(s), model(s) or theories describing the potential impact of the DAI if applicable |
| SPIRIT | 10 | Specific objectives related to benefits and harms | Specific objectives and hypotheses in relation to the DAI impact | This may not always be about the impact of the DAI, it could be in relation to the mechanisms of action | Specific objectives or hypotheses related to the DAI |
| CONSORT | 7 | Specific objectives related to benefits and harms | Specific objectives and hypotheses in relation to the DAI impact | This may not always be about the impact of the DAI, it could be in relation to the mechanisms of action | Specific objectives or hypotheses related to the DAI |
| SPIRIT | 13 | Settings (for example, community, hospital) and locations (for example, countries, sites) where the trial will be conducted | Description of how the welfare of the dog is considered in the study settings | Include study context and settings | Description of how considerations related to dog welfare are incorporated in the study context and settings |
| CONSORT | 11 | Settings (e.g., community, hospital) and locations (e.g., countries, sites) where the trial was conducted | Description of how the welfare of the dog was considered in the study settings | Include study context and settings | Description of how considerations related to dog welfare were incorporated in the study context and settings |
| SPIRIT | 13 | Settings (for example, community, hospital) and locations (for example, countries, sites) where the trial will be conducted | Description of the sensory characteristics in which the DAI takes place (e.g., noise levels, lighting, smells, flooring) | There may be other characteristics, other than sensory, that may have an impact on the dog | Description of the characteristics of study settings and the DAI that may affect the dog(s) (e.g., room size, noises, smells, lighting) |
| CONSORT | 11 | Settings (e.g., community, hospital) and locations (e.g., countries, sites) where the trial was conducted | Description of the sensory characteristics in which the DAI took place (e.g., noise levels, lighting, smells, flooring) | There may be other characteristics, other than sensory, that may have an impact on the dog | Description of the characteristics of study settings and the DAI that may affect the dog(s) (e.g., room size, noises, smells, lighting) |
| SPIRIT | 13 | Settings (for example, community, hospital) and locations (for example, countries, sites) where the trial will be conducted | Justify the suitability of the dog in relation to the social and physical environment | This is about the study context and environment | Description of how the suitability of the dog for the DAI in the given study context and environment is ensured |
| CONSORT | 11 | Settings (e.g., community, hospital) and locations (e.g., countries, sites) where the trial was conducted | Describe the suitability of the dog in relation to the social and physical environment | This is about the study context and environment | Description of how the suitability of the dog for the DAI in the given study context and environment is ensured |
| SPIRIT | 14a | Eligibility criteria for participants | Eligibility criteria relevant to interacting with the dog (e.g., absence of dog phobia, no allergies, no history of dog abuse) | Provide relevant examples without excluding population, for example say 'animal phobia' as opposed to no history of animal phobia. Also need to reflect the participant may not be interacting with the dog, but just engaging | Eligibility criteria relevant to engaging/interacting with the dog, if any (e.g., dog phobia, allergies, history of dog abuse) |
| CONSORT | 12a | Eligibility criteria for participants | Eligibility criteria relevant to interacting with the dog (e.g., absence of dog phobia, no allergies, no history of dog abuse) | Provide relevant examples without excluding population, for example say 'animal phobia' as opposed to no history of animal phobia. Also need to reflect the participant may not be interacting with the dog, but just engaging | Eligibility criteria relevant to engaging/interacting with the dog, if any (e.g., dog phobia, allergies, history of dog abuse) |
| SPIRIT | 14b | If applicable, eligibility criteria for sites and for individuals who will deliver the interventions (for example, surgeons, physiotherapists) | Describe the on- and off-site requirements in relation to integrating the DAI into the trial setting | Be clear this is the criteria for sites to ensure dog welfare requirements can be met | Description of any criteria for sites to ensure dog welfare requirements can be met |
| CONSORT | 12b | If applicable, eligibility criteria for sites and for individuals delivering the interventions (e.g., surgeons, physiotherapists) | N/A | This was added as a CONSORT item because of the 2025 update | Description of any criteria for sites to ensure dog welfare requirements can be met |
| SPIRIT | 14b | If applicable, eligibility criteria for sites and for individuals who will deliver the interventions (for example, surgeons, physiotherapists) | Selection criteria for the dog(s) involved in the intervention and justification for these | N/A | Selection criteria for the dog(s) involved in the intervention and justification for these |
| CONSORT | 12b | If applicable, eligibility criteria for sites and for individuals delivering the interventions (e.g., surgeons, physiotherapists) | Selection criteria for the dog(s) involved in the intervention and justification for these | N/A | Selection criteria for the dog(s) involved in the intervention and justification for these |
| SPIRIT | 14b | If applicable, eligibility criteria for sites and for individuals who will deliver the interventions (for example, surgeons, physiotherapists) | Eligibility criteria for trained professional(s) and/or handler(s) | Eligibility criteria not appropriate, should be about describing necessary training. This should be presented so it is relevant to all parties involved in the intervention, where appropriate. Combine with items below. | Description of relevant training and education undertaken by all parties that is pertinent to their role in the intervention (e.g., participant vs dog handler). This may include training on dog welfare, safety, and intervention specific content. |
| CONSORT | 12b | If applicable, eligibility criteria for sites and for individuals delivering the interventions (e.g., surgeons, physiotherapists) | Eligibility criteria for trained professional(s) and/or handler(s) | Eligibility criteria not appropriate, should be about describing necessary training. This should be presented so it is relevant to all parties involved in the intervention, where appropriate. Combine with items below. | Description of relevant training and education undertaken by all parties that is pertinent to their role in the intervention (e.g., participant vs dog handler). This may include training on dog welfare, safety, and intervention specific content. |
| SPIRIT | 14b | If applicable, eligibility criteria for sites and for individuals who will deliver the interventions (for example, surgeons, physiotherapists) | Description of intervention-specific training completed by dog and dog-handler teams | As above | N/A |
| CONSORT | 12b | If applicable, eligibility criteria for sites and for individuals delivering the interventions (e.g., surgeons, physiotherapists) | Description of intervention-specific training completed by dog and dog-handler teams | As above | N/A |
| SPIRIT | 14b | If applicable, eligibility criteria for sites and for individuals who will deliver the interventions (for example, surgeons, physiotherapists) | Description of dog-specific risk awareness/basic welfare training completed by the individuals involved in the intervention (e.g., participant, therapist) | As above | N/A |
| CONSORT | 12b | If applicable, eligibility criteria for sites and for individuals delivering the interventions (e.g., surgeons, physiotherapists) | Description of dog-specific risk awareness/basic welfare training completed by the individuals involved in the intervention (e.g., participant, therapist) | As above | N/A |
| SPIRIT | 15a | Intervention and comparator with sufficient details to allow replication including how, when, and by whom they will be administered. If relevant, where additional materials describing the intervention and comparator (for example, intervention manual) can be accessed | Description of dog-assisted intervention goal and content | Use the acronym to preserve journal word limits | Description of DAI goal and content |
| CONSORT | 13 | Intervention and comparator with sufficient details to allow replication. If relevant, where additional materials describing the intervention and comparator (e.g., intervention manual) can be accessed | Description of dog-assisted intervention goal and content | Use the acronym to preserve journal word limits | Description of DAI goal and content |
| SPIRIT | 15a | Intervention and comparator with sufficient details to allow replication including how, when, and by whom they will be administered. If relevant, where additional materials describing the intervention and comparator (for example, intervention manual) can be accessed | Description of the proposed duration and frequency of DAI including, where possible, justification | N/A | Description of the proposed duration and frequency of DAI including, where possible, justification |
| CONSORT | 13 | Intervention and comparator with sufficient details to allow replication. If relevant, where additional materials describing the intervention and comparator (e.g., intervention manual) can be accessed | Description of the duration and frequency of DAI including, where possible, justification | N/A | Description of the proposed duration and frequency of DAI including, where possible, justification |
| SPIRIT | 15a | Intervention and comparator with sufficient details to allow replication including how, when, and by whom they will be administered. If relevant, where additional materials describing the intervention and comparator (for example, intervention manual) can be accessed | Description of tasks and roles of each individual in the DAI team (i.e., dog, dog handler, other trained professional) including details on participant-dog interactions | N/A | Description of tasks and roles of each individual in the DAI team (i.e., dog, dog handler, other trained professional) including details on participant-dog interactions |
| CONSORT | 13 | Intervention and comparator with sufficient details to allow replication. If relevant, where additional materials describing the intervention and comparator (e.g., intervention manual) can be accessed | Description of tasks and roles of each individual in the DAI team (i.e., dog, dog handler, other trained professional) including details on participant-dog interactions | N/A | Description of tasks and roles of each individual in the DAI team (i.e., dog, dog handler, other trained professional) including details on participant-dog interactions |
| SPIRIT | 15a | Intervention and comparator with sufficient details to allow replication including how, when, and by whom they will be administered. If relevant, where additional materials describing the intervention and comparator (for example, intervention manual) can be accessed | Consideration of suitability/tailoring of DAI under Equality, Diversity and Inclusivity aspects (e.g., severity of illness, cultural background and perception of dogs) | Recognise this isn't always possible or feasible, only use the severity of illness example as this is more widely relatable to | Where possible, consideration of suitability/tailoring of DAI under EDI aspects (e.g., severity of illness) |
| CONSORT | 13 | Intervention and comparator with sufficient details to allow replication. If relevant, where additional materials describing the intervention and comparator (e.g., intervention manual) can be accessed | Consideration of suitability/tailoring of DAI under Equality, Diversity and Inclusivity aspects (e.g., severity of illness, cultural background and perception of dogs) | Recognise this isn't always possible or feasible, only use the severity of illness example as this is more widely relatable to | Where possible, consideration of suitability/tailoring of DAI under EDI aspects (e.g., severity of illness) |
| SPIRIT | 15a | Intervention and comparator with sufficient details to allow replication including how, when, and by whom they will be administered. If relevant, where additional materials describing the intervention and comparator (for example, intervention manual) can be accessed | Rationale for and description of the comparator group | N/A | Rationale for and description of the comparator group |
| CONSORT | 13 | Intervention and comparator with sufficient details to allow replication. If relevant, where additional materials describing the intervention and comparator (e.g., intervention manual) can be accessed | Rationale for and description of the comparator group | N/A | Rationale for and description of the comparator group |
| SPIRIT | 15d | Concomitant care that is permitted or prohibited during the trial | Describe whether including concomitant interactions with dogs (e.g., interactions with pet/other dogs) during the trial is permitted or controlled for | Should include 'how' and make this shorter | Description of whether and how potential concomitant human-dog interactions (e.g., with pet dogs) are controlled for |
| CONSORT | 13 | Intervention and comparator with sufficient details to allow replication. If relevant, where additional materials describing the intervention and comparator (e.g., intervention manual) can be accessed | Describe whether including concomitant interactions with dogs (e.g., interactions with pet/other dogs) during the trial was permitted or controlled for | This item does not readily apply to CONSORT and should be removed | N/A |
| SPIRIT | 15b | Criteria for discontinuing or modifying allocated intervention/comparator for a trial participant (for example, drug dose change in response to harms, participant request, or improving/worsening disease) | Criteria and processes for  discontinuing or modifying the intervention based on dog, handler and participant responses | Should be and/or - Criteria and/or processes. Remove participant responses because this is covered in the original item. | Criteria and/or processes for discontinuing or modifying the intervention based on the dog and/or handler responses |
| SPIRIT | 15b | Criteria for discontinuing or modifying allocated interventions for a given trial participant (e.g., drug dose change in response to harms, participant request, or improving/worsening disease) | Description of instruments and procedures (if any) to monitor the impact of the intervention on the dog(s) | Re-word this as extension and merge SPIRIT 15b and 17 due to overlap | N/A |
| SPIRIT | 15c | Strategies to improve adherence to intervention/comparator protocols, if applicable, and any procedures for monitoring adherence (for example, drug tablet return, sessions attended) | Strategies to improve adherence to intervention protocols, and any procedures for monitoring adherence (e.g., therapist, handler, dog owner, participant, carer) | This should be removed as not DAI specific | N/A |
| SPIRIT | 16 | Primary and secondary outcomes, including the specific measurement variable (for example, systolic blood pressure), analysis metric (for example, change from baseline, final value, time to event), method of aggregation (for example, median, proportion), and time point for each outcome | Outcome measures relevant to the proposed pathway of action of the DAI | N/A | Outcome measures relevant to the proposed pathway of action of the DAI |
| CONSORT | 14 | Prespecified primary and secondary outcomes, including the specific measurement variable (e.g., systolic blood pressure), analysis metric (e.g., change from baseline, final value, time to event), method of aggregation (e.g., median, proportion), and time point for each outcome | Outcome measures relevant to the proposed pathway of action of the DAI | N/A | Outcome measures relevant to the proposed pathway of action of the DAI |
| SPIRIT | 17 | How harms are defined and assessed (for example, systematically, non-systematically) | Observation of the impact of the intervention on the dog(s) | Should include the word 'measures' | Observations/measures relevant to the impact of the intervention on the dog(s) |
| CONSORT | 15 | How harms were defined and assessed (e.g. systematically, non-systematically) | Observation of the impact of the intervention on the dog(s) | Should include the word 'measures' | Observations/measures relevant to the impact of the intervention on the dog(s) |
| SPIRIT | 16 | Primary and secondary outcomes, including the specific measurement variable (for example, systolic blood pressure), analysis metric (for example, change from baseline, final value, time to event), method of aggregation (for example, median, proportion), and time point for each outcome | Any measures relevant to the impact of the intervention on the dog handler(s) | Remove this item, not suitably specific to DAI as it is about the impact of the intervention on the facilitator | N/A |
| CONSORT | 14 | Prespecified primary and secondary outcomes, including the specific measurement variable (e.g., systolic blood pressure), analysis metric (e.g., change from baseline, final value, time to event), method of aggregation (e.g., median, proportion), and time point for each outcome | Any measures relevant to the impact of the intervention on the dog handler(s) | Remove this item, not suitably specific to DAI as it is about the impact of the intervention on the facilitator | N/A |
| SPIRIT | 16 | Primary and secondary outcomes, including the specific measurement variable (for example, systolic blood pressure), analysis metric (for example, change from baseline, final value, time to event), method of aggregation (for example, median, proportion), and time point for each outcome | Where applicable, justify the use of unblinded outcome measures in relation to the DAI | N/A | Where applicable, justify the use of unblinded outcome measures in relation to the DAI |
| CONSORT | 14 | Prespecified primary and secondary outcomes, including the specific measurement variable (e.g., systolic blood pressure), analysis metric (e.g., change from baseline, final value, time to event), method of aggregation (e.g., median, proportion), and time point for each outcome | Where applicable, justify the use of unblinded outcome measures in relation to the DAI | N/A | Where applicable, justify the use of unblinded outcome measures in relation to the DAI |
| SPIRIT | 18 | Time schedule of enrolment, interventions (including any run-ins and washouts), assessments, and visits for participants. A schematic diagram is highly recommended | Time schedule of any required participant-dog training/familiarity sessions prior to commencement of the intervention | N/A | Time schedule of any required participant-dog training/familiarity sessions prior to commencement of the intervention |
| CONSORT | 22a | For each group, the numbers of participants who were randomly assigned, received intended intervention, and were analysed for the primary outcome | Time schedule and completion rates of any required participant-dog training/familiarity sessions prior to commencement of the intervention | N/A | Time schedule and completion rates of any required participant-dog training/familiarity sessions prior to commencement of the intervention |
| SPIRIT | 19 | How sample size was determined, including all assumptions supporting the sample size calculation | Estimated number of dog handler teams required to safely and effectively deliver an intervention | Focus more on capacity to safely deliver the intervention, more than a statistical judgement | Description of capacity of dog-handler teams required to safely and effectively deliver the intervention |
| CONSORT | 16a | How sample size was determined | How the number of dog handler teams was determined | Focus more on capacity to safely deliver the intervention, more than a statistical judgement | Description of capacity of dog-handler teams required to safely and effectively deliver the intervention |
| SPIRIT | 28b | Explanation of any interim analyses and stopping guidelines, including who will have access to these interim results and make the final decision to terminate the trial | Justification, and description, of whether interim analyses and stopping guidelines consider dog welfare | N/A | Justification, and description, of whether interim analyses and stopping guidelines consider dog welfare |
| CONSORT | 16b | Explanation of any interim analyses and stopping guidelines | Justification for whether stopping guidelines considered dog welfare | The wording should reflect that used in the SPIRIT extension | Justification, and description, of whether interim analyses and stopping guidelines consider dog welfare |
| SPIRIT | 20 | Strategies for achieving adequate participant enrolment to reach target sample size | Strategies for recruiting appropriate types and numbers of dog handler teams for the study population | Remove item due to overlap with SPIRIT 19 and CONSORT 16a | N/A |
| CONSORT | 24a | Intervention and comparator as they were actually administered (e.g., where appropriate, who delivered the intervention/comparator, whether participants adhered, whether they were delivered as intended (fidelity)) | Strategies used to recruit appropriate types and numbers of dog handler teams for the study population | Remove item due to overlap with SPIRIT 19 and CONSORT 16a | N/A |
| SPIRIT | 21b | Type of randomization (simple or restricted) and details of any factors for stratification. To reduce predictability of a random sequence, other details of any planned restriction (for example, blocking) should be provided in a separate document that is unavailable to those who enrol participants or assign interventions | Description of whether concomitant interactions with dogs (e.g., interactions with pet/other dogs) is included as a stratification variable | N/A | Description of whether concomitant interactions with dogs (e.g., interactions with pet/other dogs) is included as a stratification variable |
| CONSORT | 17b | Type of randomisation and details of any restriction (e.g., stratification, blocking and block size) | Description of whether concomitant interactions with dogs (e.g., interactions with pet/other dogs) was included as a stratification variable | Don't need to specify 'was', can keep same wording as SPIRIT | Description of whether concomitant interactions with dogs (e.g., interactions with pet/other dogs) is included as a stratification variable |
| SPIRIT | 21a | Who will generate the random allocation sequence, and the method used | Describe the process of matching the dog handler teams (dog & handler) with participants | Reflect this is not always possible or relevant and merge with the item below regarding 'who' is responsible for this | If possible, describe the process of matching dog-handler teams (dog & handler) to participants, including the individual/team responsible for this process, where relevant |
| CONSORT | 19 | Whether the personnel who enrolled and those who assigned participants to the interventions had access to the random allocation sequence | Describe the process of matching the dog handler teams (dog & handler) with participants | Reflect this is not always possible or relevant and merge with the item below regarding 'who' is responsible for this | If possible, describe the process of matching dog-handler teams (dog & handler) to participants, including the individual/team responsible for this process, where relevant |
| SPIRIT | 21a | Who will generate the random allocation sequence, and the method used | Who will select the dog handler teams (dog & handler) for the DAI | Merge this item with the one above detailing the matching process | N/A |
| CONSORT | 19 | Whether the personnel who enrolled and those who assigned participants to the interventions had access to the random allocation sequence | Who selected the dog handler teams (dog & handler) for the DAI | Merge this item with the one above detailing the matching process | N/A |
| SPIRIT | 24a | Who will be blinded after assignment to interventions (for example, participants, care providers, outcome assessors, data analysts) | Description and justification of whether trained professional(s) and dog handler(s) will be blinded to the outcomes | Change 'will be' to 'are' to keep tense neutral. Add 'and how'. | Description and justification of whether trained professional(s) and dog handler(s) are blinded to the outcomes and how |
| CONSORT | 20a | Who was blinded after assignment to interventions (e.g., participants, care providers, outcome assessors, data analysts) | Description and justification of whether trained professional(s) and dog handler(s) were blinded to the outcomes | Change 'will be' to 'are' to keep tense neutral. Add 'and how'. | Description and justification of whether trained professional(s) and dog handler(s) are blinded to the outcomes and how |
| SPIRIT | 27b | Methods for any additional analyses (e.g., subgroup and sensitivity analyses) | Explain whether a subgroup analysis considers dog characteristics (e.g., size) | N/A | Explain whether a subgroup analysis considers dog characteristics (e.g., size) |
| CONSORT | 28 | Any other analyses performed, including subgroup and sensitivity analyses, distinguishing prespecified from post hoc | Explain whether a subgroup analysis included dog characteristics (e.g. size) | Change the wording match SPIRIT | Explain whether a subgroup analysis considers dog characteristics (e.g., size) |
| SPIRIT | 28a | Composition of data monitoring committee (DMC); summary of its role and reporting structure; statement of whether it is independent from the sponsor and funder; conflicts of interest and reference to where further details about its charter can be found, if not in the protocol. Alternatively, an explanation of why a DMC is not needed | Summary of the role of the DMC or relevant committee in relation to monitoring dog welfare | Change to 'or other relevant committee' | Summary of the role of the DMC or other relevant committee in relation to monitoring dog welfare |
| SPIRIT | 29 | Frequency and procedures for monitoring trial conduct. If there is no monitoring, give explanation. | Justification for, and description of, the risk assessment and monitoring instruments used during the trial conduct | Re-word this as extension should be specific to the dog and not include any facilitators. Merge with all extensions listed for SPIRIT 29 and SPIRIT 30 due to overlap | Plans for collecting, assessing, reporting and managing harms or unintended effects for the dog(s) |
| CONSORT | 27 | All important harms or unintended effects in each group | Harms or unintended effects for all relevant parties (e.g., dog, handler, participant) associated with the DAI and how these were assessed | Re-word this as extension should be specific to the dog and not include any facilitators. Merge with all extensions listed for CONSORT 27 due to overlap | Harms or unintended effects associated with the DAI, for the dog, and how these were assessed |
| CONSORT | 27 | All important harms or unintended effects in each group | Procedures for collecting, assessing, reporting and managing harms or unintended effects for the dog(s) | As above | N/A |
| SPIRIT | 29 | Frequency and procedures for monitoring trial conduct. If there is no monitoring, give explanation | Plans for collecting, assessing,  reporting and managing harms or unintended effects for the dog handler team | As above | N/A |
| CONSORT | 27 | All important harms or unintended effects in each group | Procedures for collecting, assessing, reporting and managing harms or unintended effects for the dog handler team | As above | N/A |
| SPIRIT | 30 | Plans for seeking research ethics committee/institutional review board approval | Plans to protect and monitor dog wellbeing during the study | As above | N/A |
| CONSORT | 22b | For each group, losses and exclusions after randomisation, together with reasons | Participant losses/exclusions for reasons relating to interacting with a dog | N/A | Participant losses/exclusions for reasons relating to interacting with a dog |
| CONSORT | 22b | For each group, losses and exclusions after randomisation, together with reasons | Losses/exclusions of the dog(s)/dog handler(s) with reasons why | N/A | Losses/exclusions of the dog(s)/dog handler(s) with reasons why |
| CONSORT | 25 | A table showing baseline demographic and clinical characteristics for each group | A table showing the number and characteristics of the dog(s) involved | This may not be presented in a table | Description of the number and characteristics of the dog(s) and their handler(s) involved |
| CONSORT | 25 | A table showing baseline demographic and clinical characteristics for each group | A table showing the number and characteristics of dog handler teams involved | Combine with item above | N/A |
| CONSORT | 25 | A table showing baseline demographic and clinical characteristics for each group | Baseline data indicating concomitant interactions with dogs (e.g., interactions with pets/other dogs) if relevant | N/A | Baseline data indicating concomitant interactions with dogs (e.g., interactions with pets/other dogs) if relevant |
| SPIRIT | 34 | Provisions, if any, for ancillary and post-trial care, and for compensation to those who suffer harm from trial participation | Description for post-intervention care in relation to dog wellbeing where applicable | This should include post-session care | Description for post-session and/or post-intervention care in relation to dog wellbeing where applicable. |
| SPIRIT | 34 | Provisions, if any, for ancillary and post-trial care, and for compensation to those who suffer harm from trial participation | Provisions for supporting and managing the end of interactions between the dog and participant | N/A | Provisions for supporting and managing the end of interactions between the dog and participant (post-session and/or post-intervention) |
